# Rational design using sequence information only produces a peptide that binds to the intrinsically disordered region of p53

**DOI:** 10.1038/s41598-019-44688-0

**Published:** 2019-06-28

**Authors:** Kiyoto Kamagata, Eriko Mano, Yuji Itoh, Takuro Wakamoto, Ryo Kitahara, Saori Kanbayashi, Hiroto Takahashi, Agato Murata, Tomoshi Kameda

**Affiliations:** 1 Institute of Multidisciplinary Research for Advanced Materials, Tohoku University, Katahira 2-1-1, Aoba-ku, Sendai, 980-8577 Japan; 2 Department of Chemistry, Graduate School of Science, Tohoku University, Sendai, 980-8578 Japan; 3 Graduate School of Life Sciences, Ritsumeikan University, Kusatsu, 525-8577 Japan; 4 College of Pharmaceutical Sciences, Ritsumeikan University, Kusatsu, 525-8577 Japan; 5 Artificial Intelligence Research Center, National Institute of Advanced Industrial Science and Technology (AIST), Koto, Tokyo 135-0064 Japan

**Keywords:** Intrinsically disordered proteins, Fluorescence imaging, DNA-binding proteins, Virtual screening

## Abstract

Intrinsically disordered regions (IDRs) of proteins are involved in many diseases. The rational drug design against disease-mediating proteins is often based on the 3D structure; however, the flexible structure of IDRs hinders the use of such structure-based design methods. Here, we developed a rational design method to obtain a peptide that can bind an IDR using only sequence information based on the statistical contact energy of amino acid pairs. We applied the method to the disordered C-terminal domain of the tumor suppressor p53. Titration experiments revealed that one of the designed peptides, DP6, has a druggable affinity of ~1 μM to the p53 C-terminal domain. NMR spectroscopy and molecular dynamics simulation revealed that DP6 selectively binds to the vicinity of the target sequence in the C-terminal domain of p53. DP6 inhibits the nonspecific DNA binding of a tetrameric form of the p53 C-terminal domain, but does not significantly affect the specific DNA binding of a tetrameric form of the p53 core domain. Single-molecule measurements revealed that DP6 retards the 1D sliding of p53 along DNA, implying modulation of the target searching of p53. Statistical potential-based design may be useful in designing peptides that target IDRs for therapeutic purposes.

## Introduction

Intrinsically disordered regions (IDRs) of proteins lack a defined 3D structure. Approximately 10–35% of prokaryotic and 15–45% of eukaryotic proteins are estimated to contain IDRs^1^. IDRs function as hubs in protein-protein interaction networks, the regulation of transcription and signaling pathways, and phase separation. IDRs are involved in many diseases and are considered drug targets^2–4^. The major drug design targeting IDR is based on the experimental screening of chemicals^4^. By contrast, structure-based drug design, which is one of the most commonly used methods to design drugs targeting proteins, is difficult to apply to IDRs due to their flexible structure^4^. A unique successful method of the rational drug design targeting IDRs is virtual screening of chemicals that bind pockets formed in IDR conformations^5,6^. Before the screening, pockets of the IDR need to be estimated by MD simulation or NMR. Accordingly, a rational design approach without 3D structure information is required for targeting IDRs.

p53, an intrinsically disordered protein containing IDRs, is a multifunctional transcription factor that can suppress cell tumorigenesis and is a desirable drug target^7,8^. Fifty percent of the gene mutations identified in tumor cells occur in p53 gene^9^. p53 is composed of an N-terminal (NT) domain, a core domain, a linker, a tetramerization domain, and a C-terminal (CT) domain. The core and tetramerization domains form a specific tertiary structure, while the other two domains and the linker are IDRs. The core and CT domains are involved in the specific and nonspecific DNA binding, respectively. The 1D sliding of p53 along DNA is essential to searching for the target site among tremendous amounts of nonspecific DNA^10^. p53 possesses two sliding modes with a different conformation^11–13^. The CT domain facilitates the 1D sliding of p53^11,14^ and regulates the transcription of downstream genes^15–18^. Several studies focused on drug design or function modification for p53^8,17,19–27^ have mainly targeted the folded domains, and successful drug design for the IDRs of p53 has been limited to two studies^28,29^. Gabizon *et al*. identified several peptides that bind to the tetrameric form of the disordered CT domain of p53 by screening peptides derived from natural proteins bound to p53^29^.

Peptides are promising drug candidates^30,31^ and may work for targeting IDRs^29^. The combination of 20 amino acids with different characteristics has the potential to generate peptides with high affinity for the target IDR. Also, the flexible peptide can fit any conformation of the IDR. We need to search for peptides with a high affinity for the target IDR among an enormous number of candidates; for example, for a 16-residue peptide, 20^16^ candidates are possible. The theoretical pool of peptides is very large, while using peptides from natural proteins limits the number of peptides that can be tested, hence it is needed to develop a computer-based method for drug design.

Here, we aimed to develop the design method of peptides that can bind IDRs and then apply the method to the p53 IDR. The method uses only the IDR’s sequence information without considering its structure. For the design, we used the Miyazawa-Jernigan (MJ) potential, which reflects the residue-residue potential for favorable and unfavorable contacts based on the analysis of known protein crystal structures in protein crystal structures^32^. MJ potential has been used in protein folding simulation and structural prediction^33–37^. As a target IDR, we chose the CT domain of p53. We designed six peptides, and found that three peptides bound to the CT domain tighter than the peptides identified by Gabizon *et al*.^29^. A series of experiments including NMR, molecular dynamics (MD) simulations, ensemble titration, and single-molecule fluorescence demonstrated that one designed peptide with the highest affinity can modulate the nonspecific DNA binding of p53 and the 1D sliding of p53 along DNA. This is the first approach to target IDRs using only their sequence information and is potentially applicable to many disease-related proteins containing IDRs.

## Results

### MJ potential-based peptide design identifies a peptide with micromolar affinity for the p53 CT domain

**Figure 1:**
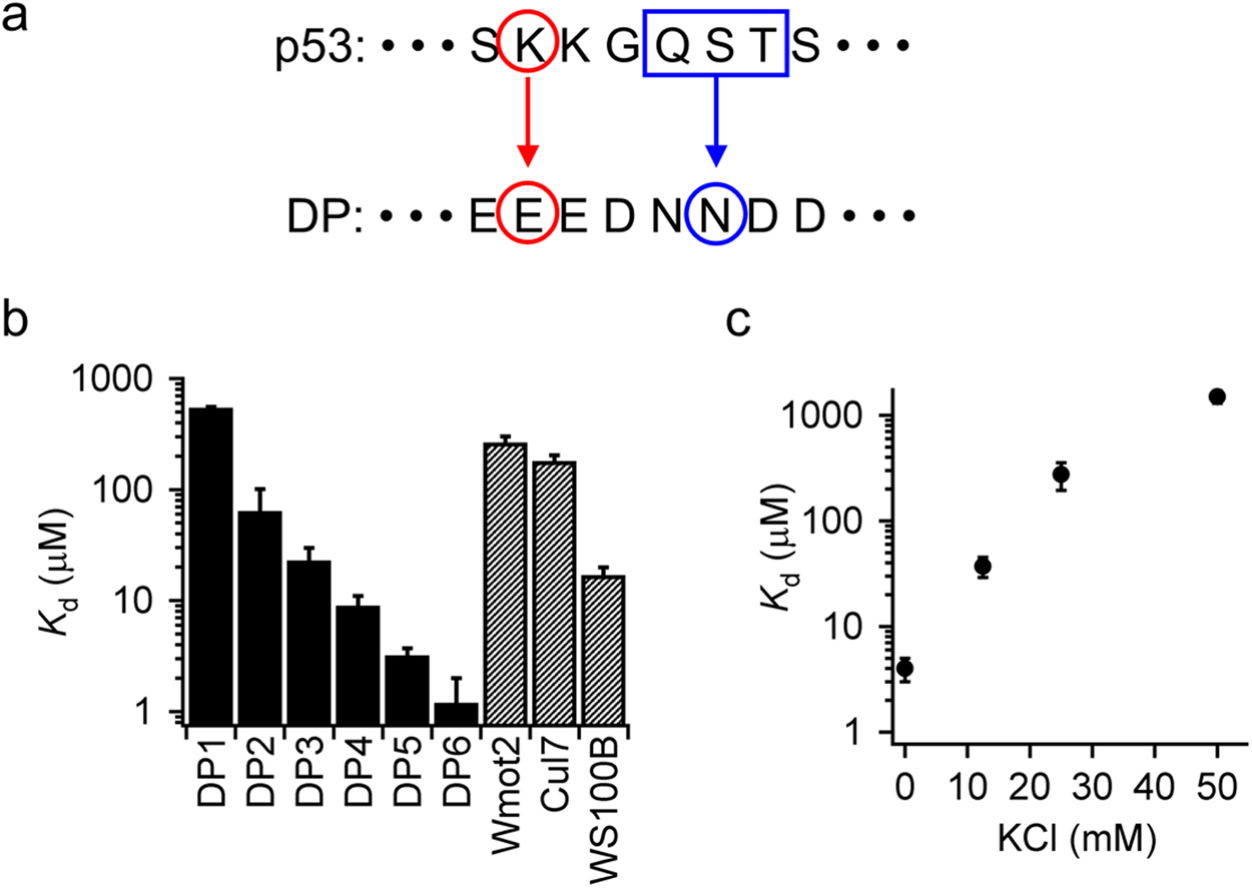
Binding of designed peptides to the monomeric CT domain of p53. (**a**) Schematic diagram of peptide design. Red and blue indicate one by one residue and one by three residue design, respectively. DP, designed peptide. (**b**) Dissociation constants of designed peptides (DP1–DP6) or peptides from natural proteins (Wmot2, Cul7, and WS100B) for the CT peptide of p53 in the absence of salt. (**c**) Salt dependence of the dissociation constant of DP6 with the CT peptide. In panels (b,c), the error is the SEM of the fitting.

Using MJ potential^32^, we designed peptide sequences to bind the disordered CT domain of p53. Specifically, six peptides with 13 or 16 residues were designed to minimize the total statistical binding energy for one by one residue (designed peptides DP1–4) or one by three residues including two adjacent residues (designed peptides DP5–6) (Supplementary Figure S1 and Table 1). One by one assumes the contacts of two residues facing in the two sequences, while one by three assumes the contacts to three sequential residues (Fig. 1a). The binding energy between the *i*th residue of the CT domain and the *j*th residue of a designed peptide was calculated as *e*_*ij*_ + *e*_*rr*_ − *e*_*ir*_ − *e*_*jr*_, as defined by Miyazawa and Jernigan^32,38^, where *e*_*ij*_ denotes the energy difference between the formation of contacts between the *i*th and *j*th residues, and the same residues exposed to solvent. The notation *r* represents averaging over all amino acids. To test how well our method worked, we titrated the designed peptides against the CT peptide (residues 367–393) labeled with a fluorescent dye, FAM, using a fluorometer with fluorescence anisotropy^11^ (Supplementary Fig. S2a). All titration curves were well fitted with equations 1 and 2 (see Methods) based on one by one binding. The apparent dissociation constant of the designed peptides (*K*_D_) with the CT peptide ranged from 1.2 ± 0.8 μM to 550 ± 10 μM in the absence of salt, and DP6 showed the strongest binding to the CT domain (Fig. 1b and Table 1). One by three design (DP5–6) improved the binding ability of the designed peptide to the CT peptide by at least six-fold compared to one by one design (DP1–4). The affinity of the designed peptides for the CT peptide correlated with the estimated binding energy (Supplementary Fig. S3). These results suggest that interactions between residues that are favorable in natural folded proteins can stabilize complexes involving disordered proteins.

**Table 1:** Length, sequence, and dissociation constant of peptides used in this study.

Peptide	L*	Sequence	*K*_d_ (μM)^†^
DP1	13	EHEEIFMESKGWR	60 ± 40
DP2	13	EDEEGNDSDEHEE	23 ± 7
DP3	16	DEHEEIFMESKGWRDR	550 ± 10
DP4	16	DHIEDEEGNDSDEHEE	9 ± 2
DP5	13	EEEEDNNDDDEEE	3.2 ± 0.5
DP6	16	EEEEEDNNDDDEEEWF	1.2 ± 0.8
Wmot2 (266–280)^‡^	16	WSTNGDTFLGGEDGDQ	270 ± 40
Cul7 (386–400)^‡^	15	LDDYEEISAGDEGEF	180 ± 20
WS100B (61–75)^‡^	16	WLDNDGDGECDFQEFM	17 ± 3

*Residue length. ^†^*K*_d_ was determined by titrating peptides against the CT peptide of p53 in the absence of salt. The error of *K*_d_ is the SEM of the fitting. ^‡^The peptides were identified from natural proteins^29^.

Next, we compared the affinity of our designed peptides to that of peptides derived from natural p53-binding proteins and confirmed to bind to the CT peptide in an earlier report^29^. To this end, we titrated three natural peptides against the CT peptide under the same conditions and determined the *K*_D_ (Fig. 1b and Supplementary Fig. S2b). The *K*_D_ of DP6 with respect to the CT domain was at least 12 times stronger than the *K*_D_ of the natural peptides (Table 1).

To elucidate the mechanism of DP6 binding to the CT domain, we examined the dependence of *K*_D_ on salt concentration (Fig. 1c). *K*_D_ decreased as the K^+^ concentration increased, suggesting that the binding was mainly governed by electrostatic interactions between the +6 net charge of DP6 and the −4 net charge of the CT peptide. The circular dichroism spectra of the DP6–CT peptide complex and of the DP6 and CT peptides alone showed no significant secondary structure, implying disordered-disordered contacts in the complex (Supplementary Fig. S4).

### DP6 binds selectively to the target region and its vicinity via electrostatic and hydrophobic interactions

**Figure 2:**
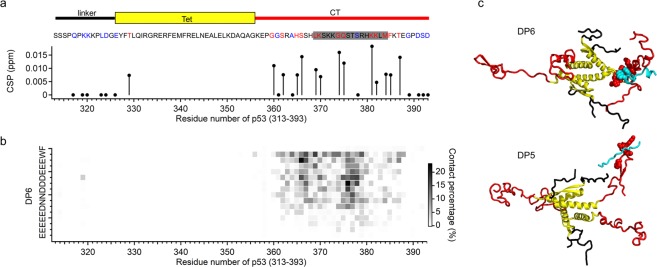
Binding of the designed peptide DP6 to tetrameric p53 (313–393). (**a**) Chemical shift perturbations (CSP) of the ^1^H-^15^N HSQC spectrum of p53 (313–393) in the absence/presence of DP6. Red and blue characters represent residues showing positive or no CSP, respectively. Gray highlighting indicates the target sequence of p53 used for the design of DP6. (**b**) Contact map between residues of DP6 and p53 in MD simulation. (**c**) Typical snapshots of DP6 and p53 (313–393) tetramer complex in MD simulation. Cyan, black, yellow, and red denote designed peptide, linker, the Tet domain, and the CT domain of p53, respectively. Hydrophobic amino acids of the CT domain, DP5, and DP6 in contacts are depicted in space-filling representation.

To examine whether DP6 binds specifically to the designed target position of p53, we conducted NMR analysis of a ^15^N-labeled p53 (residues 313–393) tetramer including linker, Tet, and CT domains in the absence and presence of DP6 (Fig. 2a and Supplementary Fig. S5). Clear chemical shift perturbations in the ^1^H-^15^N HSQC spectrum upon binding to DP6 were observed in residues 329, 360, 362, 365, 366, 369, 370, 374, 375, 381, 382, 384, 385, and 387 of p53. These residues correspond to the designed target of the CT domain (residues 369–384) for DP6 and its vicinity. To further investigate the complex structure, we conducted MD simulations of the p53 (313–393) tetramer and DP6. The contact map showed significant interaction between residues 360–385 of p53 and DP6, which is consistent with the chemical shift perturbations (Fig. 2a,b). Furthermore, the identified binding site of DP6 agreed with the p53 sequence region having lower MJ binding energy for DP6 (Supplementary Fig. S6). In the MD simulation trajectory, the DP6 and CT domains showed flexibility, and no specific tertiary structure was formed (Supplemental Movie). In these structures, contacts between the acidic residues of DP6 and the basic residues of the CT domain were always observed. In addition, W15 and F16 in DP6 and hydrophobic regions in the CT domain (especially, residues 361–369, 371, 373–379, 385, 387, 388) always formed a hydrophobic cluster (Fig. 2c). These results suggest that electrostatic and hydrophobic interactions strongly stabilized the DP6–CT domain complex compared with other peptides. In fact, such hydrophobic interactions were not observed between the CT domain and DP5, which may explain the relatively weak affinity compared with that of DP6 (Fig. 2c). Accordingly, we conclude that DP6 binds selectively to the designed target region and its vicinity in the CT domain of p53.

### DP6 interferes with nonspecific DNA binding of p53

**Figure 3:**
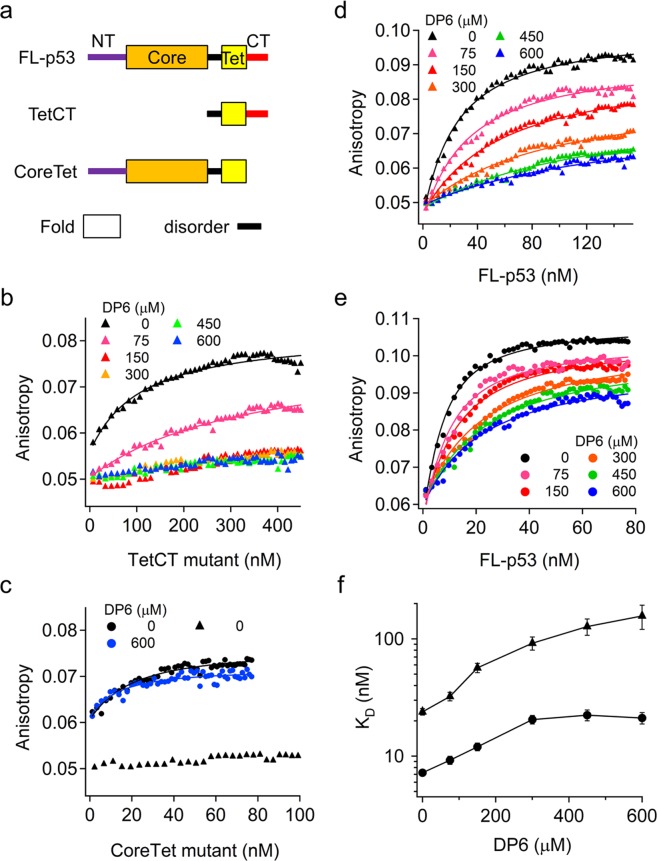
Effect of the designed peptide DP6 on the DNA binding of the p53 tetramer. (**a**) p53 constructs used in this study. NT, core, Tet, and CT represent the N-terminal, core, tetramerization, and C-terminal domains of p53, respectively. Thick and thin primary structures correspond to folded and disordered regions, respectively. (**b**) Titration of the TetCT mutant against nspDNA at various DP6 concentrations. (**c**) Titration of the CoreTet mutant against spDNA in the presence (blue circles) and absence (black circles) of 600 μM DP6 and against nspDNA in the absence of DP6 (triangles). (**d**) Titration of FL-p53 against nspDNA at various DP6 concentrations. (**e**) Titration of FL-p53 against spDNAat various DP6 concentrations. (**f**) Affinity of FL-p53 for nspDNA (triangles) and spDNA (circles) at various DP6 concentrations. The errors were the SEM of the fitting. In panels (**b**–**e**), tetramer concentrations are used for the p53 mutants, and the solid curves are the best-fitted curves using Equations (1) and (2) described in the Methods.

To examine the effect of DP6 on the DNA binding of p53, we prepared three p53 constructs: full-length p53 (FL-p53), and two mutants each containing one of its two DNA binding domains, a tetrameric form of the CT domain (TetCT mutant), and a tetrameric form of the core domain (CoreTet mutant) as reported previously^12^ (Fig. 3a). For the p53 mutants, we used a thermostabilized form of p53 with a single exposed cysteine, which is suitable for *in vitro* ensemble and single-molecule studies^11^.

To examine the effect of DP6 on the affinity of the TetCT mutant for DNA, we titrated TetCT mutant against nonspecific DNA (nspDNA) labeled with 6-FAM at 0–600 μM DP6 based on fluorescence anisotropy^11^. The solution used here included 100 mM KCl to mimic physiological conditions. In the absence of DP6, the TetCT mutant bound to nspDNA (*K*_D_ = 110 ± 20 nM for tetramer). The anisotropy change for nspDNA was repressed as the DP6 concentration increased (Fig. 3b). However, no significant change in anisotropy was observed at more than 150 μM DP6, suggesting that DP6 inhibits the binding of the TetCT mutant to nspDNA by competitive binding to the CT domain of the TetCT mutant. For control experiments investigating the effect of DP6 on the binding of the core domain to specific DNA (spDNA), we titrated the CoreTet mutant against spDNA in the presence/absence of 600 μM DP6 (Fig. 3c, circles). DP6 did not weaken the binding of the CoreTet mutant to spDNA (Supplementary Table S1). Nonspecific binding of the CoreTet mutant was not observed, confirming that DNA binding of the CoreTet mutant in the presence of DP6 was specific (Fig. 3c, triangles). Accordingly, the results suggest that DP6 binds specifically to the CT domain of p53 and prevents nonspecific binding to DNA, thereby competitively maintaining the association of the core domain with spDNA.

We next examined the effect of DP6 on the affinity of FL-p53 to DNA by titrating FL-p53 against nspDNA and spDNA at 0–600 μM DP6. The anisotropy change for nspDNA was repressed as the DP6 concentration increased, indicating that the nonspecific affinity of FL-p53 was weakened by DP6 (Fig. 3d). The anisotropy change for spDNA was also repressed by DP6 (Fig. 3e), but the repression was more effective for nspDNA than for spDNA. The *K*_D_ of FL-p53 for nspDNA gradually increased with the addition of DP6 and was 7-fold higher at 600 μM than at 0 μM DP6 (Fig. 3d and Supplementary Table S1). By contrast, the *K*_D_ for spDNA increased to 3-fold and was saturated at 300 μM DP6 (Fig. 3e and Supplementary Table S1). In contrast to DP6, DP5 did not affect the affinity of FL-p53, suggesting that W and/or F of DP6 strengthens the binding of the designed peptide to FL-p53 (Supplementary Fig. S7 and Supplementary Table S2). These results demonstrate that DP6 weakens the nonspecific DNA binding of FL-p53 more efficiently than the specific DNA binding.

### DP6 retards 1D sliding of p53 along DNA

**Figure 4:**
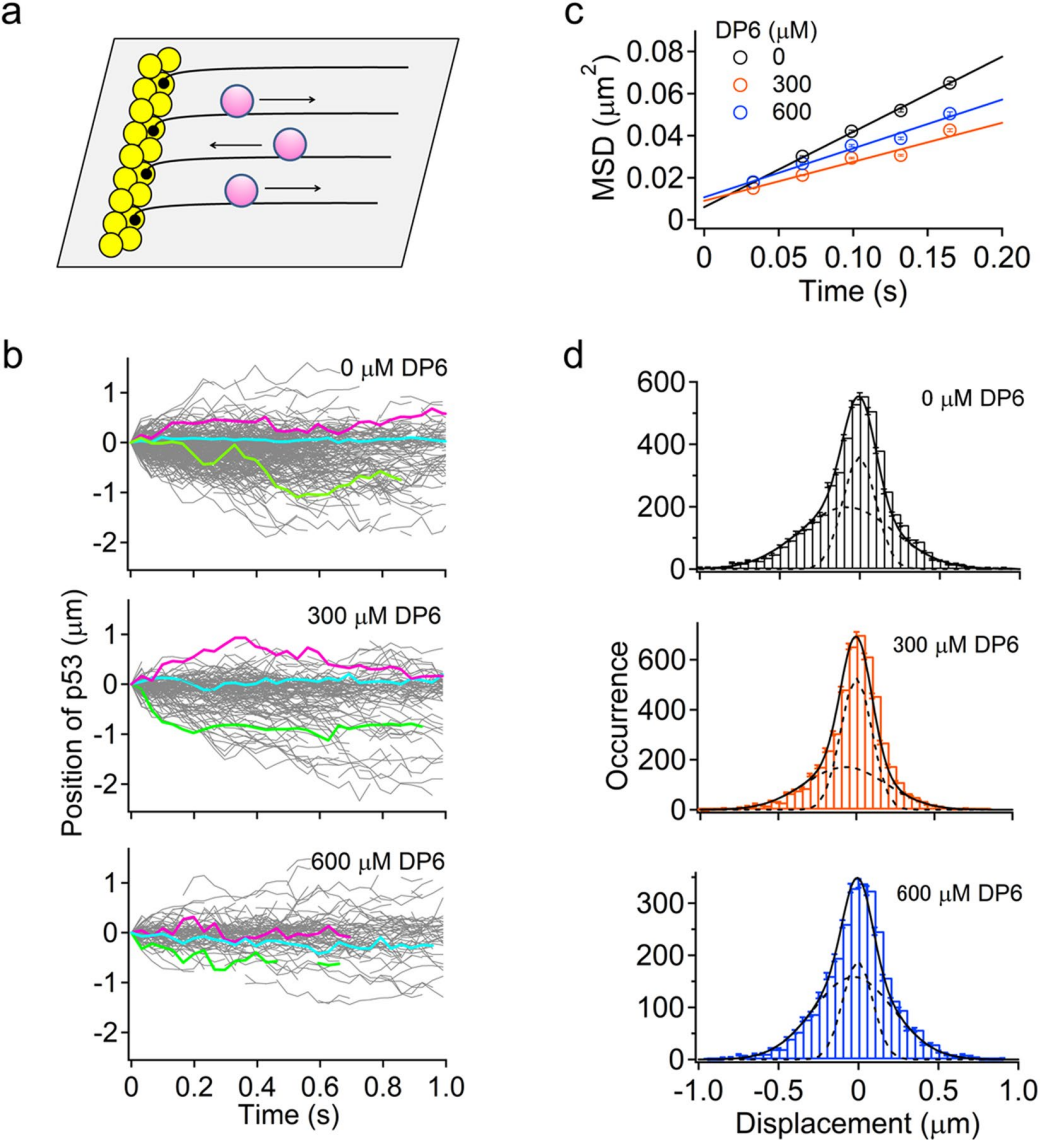
Effect of the designed peptide DP6 on 1D sliding of the p53 tetramer along DNA. (**a**) Schematic diagram of the single-molecule detection of p53 labeled with Atto532 on a DNA array. Yellow circles, pink circles, and solid lines represent NeutrAvidin, p53, and DNA, respectively. (**b**) Typical single-molecule trajectories of FL-p53 along nonspecific DNA at various DP6 concentrations. Several traces are colored for clarity. (**c**) Time courses of the averaged MSD of FL-p53 at various DP6 concentrations. (**d**) Displacement distributions for the sliding dynamics of FL-p53 at various DP6 concentrations. Bars represent the displacement distributions observed at time intervals of 165 ms. Errors of the bar were estimated by a bootstrap method with 1,000 iterations. Solid and dashed curves are best-fitted curves obtained using Equation (3) described in Methods and the distributions of each mode, respectively.

To test whether DP6 affects the 1D sliding of p53 along DNA, we visualized the 1D sliding of FL-p53 labeled with a fluorescent dye, Atto532, along nspDNA at 0, 300, and 600 μM DP6 using single-molecule fluorescence microscopy coupled with the DNA array “DNA garden”^10,11,39^ (Fig. 4a). We conducted the measurements within 50 min after the dilution of stock solution under conditions in which p53 maintains a tetrameric form^40^ and obtained 158–290 trajectories in various DP6 concentrations (Fig. 4b). The mean square displacement (MSD) plots of FL-p53 were linear in all conditions, indicating the diffusional motion of p53 along DNA at various DP6 concentrations (Fig. 4c). The average diffusion coefficient, calculated from the slope of MSD, decreased to 0.5-fold at 300 μM DP6 and 0.6-fold at 600 μM (Supplementary Table S3). These results demonstrate that DP6 retards the 1D sliding of p53 along DNA.

To examine how DP6 affects the two sliding modes of p53, we analyzed the displacement distributions of p53 at the time interval of 165 ms in different DP6 concentrations. All displacement distributions were well fitted with the sum of two Gaussian functions, which assumes two diffusional motions (Eq. 3, see Methods), indicating that FL-p53 possesses two sliding modes in the presence of DP6. The diffusion coefficient of the fast mode decreased to 0.8-fold at 300 μM DP6 and to 0.7-fold at 600 μM, thereby reducing the average diffusion coefficient (Supplementary Table S3). By contrast, no significant change in the slow mode is consistent with the tight interaction of the core domains with DNA identified in the slow mode^12^. Accordingly, the fast mode of FL-p53 sliding, rather than the slow mode, is retarded upon the association of DP6 with FL-p53.

## Discussion

In this study, we developed the design method of peptides that can bind IDRs using only the IDR’s sequence information without its structural information. To test this method, we targeted the disordered CT domain of p53. We found that DP6 has the high affinity for the CT domain, and modulates the affinity of p53 for DNA and the target search. These results suggest that our design method has a potential to generate druggable peptide candidates for IDRs. Here, we discuss the mechanism for action of DP6 and the comparison of drug design methods for IDRs.

**Figure 5:**
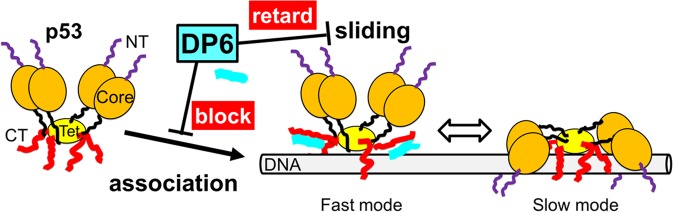
Model for regulation of the affinity and 1D sliding of p53 by the designed peptide DP6. DP6 (cyan) weakens the association of p53 with DNA (gray bar) and retards the diffusion of the fast mode of p53 along DNA upon association with the CT domain of p53 (red). Purple, orange, and yellow represent the NT, core, and Tet domains of p53, respectively. The structure of the p53–DNA complex is described based on the results of our previous study^12^.

We propose a mechanism for how DP6 affects p53 (Fig. 5). One of the actions of DP6 is to competitively block the association of the CT domain of p53 with nonspecific DNA. Previous spectroscopic studies including chemical shift perturbations in NMR and the effects of mutation on the affinity revealed that residues 365−382, including lysine residues 372, 373, 381, and 382 in the CT domain, interact with DNA^41,42^. The NMR data in this study demonstrate that DP6 interacts with residues 360–387 of p53 (Fig. 2a). Therefore, it is likely that DP6 sterically prevents the association of the CT domain with DNA, reducing the affinity of p53 for nonspecific DNA (Fig. 3b). This finding is supported by the fact that additives such as small nucleotides and antibodies strengthen specific affinity by blocking the CT domain^43^. Before the formation of the p53–DNA complex, the CT domain is blocked by DP6 from association with nonspecific DNA.

The other action of DP6 is to retard 1D sliding along DNA. Once the CT domain binds to DNA instead of DP6, the p53–DP6 complex slides along DNA (Fig. 5). DP6 mainly affects the fast mode in which the CT domain of p53 interacts with nonspecific DNA without core domain–DNA interaction^12^ (Fig. 4d). The observed reduction in the diffusion coefficient in the fast mode may be attributed to the larger molecular size of the p53–DP6 complex and/or intercalation with DNA by DP6. The larger size of p53 may be caused by the detachment of the CT domain from DNA and the association of DP6 with p53 and should slow the 1D sliding along DNA because of the dependence of the 1D sliding on molecular size^44,45^. As observed in DNA glycosylase^46^, tryptophan in DP6 may insert between DNA bases during the sliding of the p53–DP6 complex and thereby retard the sliding. By contrast, DP6 does not affect the slow mode, in which p53 reads the DNA sequence and recognizes the target, because in the slow mode, the core domain and CT domain interact with DNA^12^. This result confirms the specific DNA binding ability of p53. Thus, DP6 may act as a potential inhibitor of p53 by preventing the target search kinetically but not the target binding itself.

The MJ-potential-based approach has unique characteristics comparing with other approaches in the drug design targeting IDRs. Successful approach in the drug design for IDRs is the experimental or virtual screening of chemicals^4^. The experimental screening does not use any information of the IDR sequence and structure, but requires a high cost and consumes time. As in the case of p53, a small chemical RITA was discovered to bind disordered NT domain of p53 by using the experimental screening^28^. In the virtual screening, the IDR pockets are identified using MD or NMR and then the chemicals that can bind the pockets are screened^4^. The virtual screening may not work in the case of no pockets identified in the IDR conformation ensemble. By contrast, our method may be applicable to the IDRs without pocket structures, because the structural information is not used. The MJ-based approach can be applied to the IDRs with known primary sequence. In fact, the designed peptide DP6 identified in this study can bind the CT domain of p53 much stronger than the peptides from natural proteins (Table 1) and the affinity of DP6 is comparable to that of drug candidates discovered in other disordered proteins^4^. This suggests the successful searching for the druggable peptide among an enormous number of candidates. The virtual screening of peptides based on the MJ contact energy is simple and does not need a supercomputer. Accordingly, the MJ-based approach provides an alternative strategy of drug design targeting IDRs and may apply to many disease-related proteins containing IDRs.

## Materials and Methods

### Calculation of contact energy

The binding energy between the *i*th residue of the CT domain and the *j*th residue of the designed peptide, *E*, for one by one residue design was calculated as *e*_*ij*_ + *e*_*rr*_ − *e*_*ir*_ − *e*_*jr*_, as defined by Miyazawa and Jernigan^32^. The notation *r* represents averaging over all amino acids. For *e*_*ij*_, *e*_*rr*_, *e*_*ir*_, and *e*_*jr*_, we used the values reported by Miyazawa and Jernigan^32^. We calculated the binding energy by replacing the *j*th residue of a designed peptide with each of the 20 amino acids, and then determined the amino acid that gave the lowest binding energy. This procedure was repeated to obtain the sequence of a 13-residue or 16-residue peptide, and the total binding energy was calculated by summing each binding energy. A designed peptide with minimal total energy was selected among peptides designed for different initial residues of the CT domain. In addition, we selected peptides designed for different regions of the CT domain, but not with the minimum total energy. For the calculation involving one by three residues, including two adjacent residues, we added the binding energy between adjacent residues of the CT peptide and each residue of a designed peptide.

### p53 mutants and peptides

We prepared FL-p53, TetCT mutant, and CoreTet mutant as described previously^11^. For FL-p53, the thermostable and cysteine-modified mutant of human p53 (C124A, C135V, C141V, W146Y, C182S, V203A, R209P, C229Y, H233Y, Y234F, N235K, Y236F, T253V, N268D, C275A, C277A, K292C) was used^11^. The TetCT mutant corresponds to residues 293−393 of human p53 with an additional N-terminal cysteine^11^. The CoreTet mutant corresponds to residues 1–363 of FL-p53^12^. Expression and purification of the three mutants was conducted as previously described^11,12^. Briefly, all mutants with a GST tag were expressed in *Escherichia coli* and were collected from a GST column after cleavage of the GST tag and further purified by using a heparin column. The DNA binding ability of all mutants was confirmed by titration experiments as described elsewhere^10–12^. For NMR analysis, the p53 gene corresponding to residues 313–393 of human p53 in pGEX-6P-1 was generated using a KOD-Plus-Mutagenesis Kit (TOYOBO, Osaka, Japan). ^15^N-labeled p53 (313–393) was expressed in BL21 (DE3) plysS in ^15^N M9 media at 16 °C for 18 h after the addition of 0.5 mM IPTG and purified as described above^12^. For titration experiments, CT peptide (residues 367–393 of human p53) labeled with FAM at the N-terminus, designed peptides, and peptides from natural proteins were synthesized without caps and obtained with at least 95% purity (Toray Research Center Inc., Tokyo, Japan).

### NMR spectroscopy

^1^H/^15^N HSQC experiments were performed at 5 °C using a ^1^H 600 MHz NMR spectrometer (DRX-600; Bruker, Billerica, MA, USA). The solution contained 0.5 mM ^15^N-labeled p53 (313–393), 0 or 20 μM DP6, 10 mM HEPES, and 10% ^2^H_2_O at pH 7.0. HSQC cross-peaks were assigned to individual amide groups with reference to the assignments of p53 (313–393)^41^. Spectral analysis was performed using the software Topspin 1.1 (Bruker, Billerica, MA, USA) and NMRViewJ^47^.

### MD simulation

A tetramer of p53 (313–393) and the DP6 peptide were simulated using Amber16 simulator^48^ with the AMBER ff99SB force field^49^ and Generalized Born energy for solvation^50^. For the initial structure of p53 (313–393), the tetramerization domain and the missing disordered region were generated using PDB code 1OLH and modeled in PyMol software, respectively. The initial structure of DP6 was generated in extended form. Initially, DP6 was located at six positions on the *x*-, *y*-, and *z*-axes ± 100 Å from the center of mass of the p53 (313–393) tetramer as described in^51^. For system relaxation, 20 ns simulations were conducted with constraint of the p53 tetrameric domains while decreasing the temperature from 1000 to 280 K. Then, four 100 ns simulations without any constraint were conducted starting from each initial conformation with randomized initial velocity at 280 K. The temperature was controlled using a Langevin thermostat. The solvent viscosity of water was set to 1.0 ps^−1^. Noncovalent interactions were used without cutoffs. The covalent bonds of hydrogen atoms in p53 and DP6 were constrained using the SHAKE method^52^, and the integration time step was 2 fs. For the contact map, 80–100 ns conformations were used. A contact was defined as a distance of less than 6.5 Å between the centers of two side chains except for Gly, where C_α_ was used^38^.

### Titration experiments

The fluorescence anisotropy of fluorescent CT peptide or DNA was measured at 25 °C using a fluorescence spectrometer (FP-6500, JASCO Co., Tokyo, Japan) with an automatic titrator and home-build autorotating polarizer^11^. To measure the affinity of the designed peptides to the CT peptide, nonlabeled designed peptides or peptides from natural proteins^29^ were titrated into a solution containing 5 nM FAM-labeled CT peptide, 20 mM HEPES, 0.5 mM EDTA, 1 mM DTT, and 0.2 mg/mL BSA (pH 7.9). To examine the salt-dependence of the binding of DP6 to CT peptide, KCl was also added. To measure the affinity of p53 mutants to DNA, nonlabeled p53 mutants were titrated into a solution including 5 nM 6-FAM-DNA, 20 mM HEPES, 0.5 mM EDTA, 1 mM DTT, 0.2 mg/mL BSA, 100 mM KCl, 2 mM MgCl_2_, and various concentrations of DP6 (pH 7.9). spDNA and nspDNA were 30-bp sequences of the *p21* gene and a random sequence, respectively, as described elsewhere^11^ (Sigma-Aldrich Co., Tokyo, Japan). The titration curves were fitted by the following equations:

1 $${r}_{{\rm{obs}}}={r}_{{\rm{A}}}\frac{({c}_{{\rm{A}}}-{c}_{{\rm{AB}}})}{{c}_{{\rm{A}}}}+{r}_{{\rm{AB}}}\frac{{c}_{{\rm{AB}}}}{{c}_{{\rm{A}}}},$$

2 $${c}_{AB}=\frac{({c}_{{\rm{A}}}+{c}_{{\rm{B}}}+{K}_{D})-\sqrt{{({c}_{{\rm{A}}}+{c}_{{\rm{B}}}+{K}_{D})}^{2}-4{c}_{{\rm{A}}}{c}_{{\rm{B}}}}}{2},$$

where *r*_obs_, *r*_A_, *r*_AB_, *K*_D_, *c*_A_, and *c*_B_ are the observed anisotropy, anisotropy of free molecule A, anisotropy of the complex between molecules A and B, dissociation constant, total concentration of molecule A and total concentration of molecule B, respectively. For p53 mutants, the concentration was calculated for the tetramer.

### Single-molecule measurements of p53 mutants using the DNA garden method

The 1D sliding of FL-p53 labeled with Atto532 along DNA was measured by a custom-built TIRF microscope, as described previously^11,39^. The λDNA array tethered on the coverslip of the flow cell was constructed using microcontact printing as described previously^39^. FL-p53 was labeled with Atto532 as described elsewhere^11^. FL-p53 with Atto532 at 2–4 nM was introduced into the flow cell with the DNA array by a syringe pump and measured at a flow rate of 500 μL/min, corresponding to 90% extension of λDNA. The solution contained 20 mM HEPES, 0.5 mM EDTA, 1 mM DTT, 0.2 mg/mL BSA, 1 mM DTT, 2 mM Trolox, 100 mM KCl, 2 mM MgCl_2_, and 0–600 μM DP6 at pH7.9. The experiments were conducted immediately after the dilution of FL-p53 from the stock solution at more than 10 μM and finished within 50 min to prevent the dissociation of the tetramer^40^. The analysis was described previously^11^. For distribution analysis, the following fitting equation was used:

3 $$P({\rm{\delta }}x)=\sum _{i=1}^{2}\frac{{A}_{i}}{\sqrt{4{\rm{\pi }}{D}_{i}{\rm{\delta }}t}}\exp (-\frac{{({\rm{\delta }}x+{v}_{i}{\rm{\delta }}t)}^{2}}{4{D}_{i}{\rm{\delta }}t}),$$

where *P*(δ*x*), δ*t*, δ*x*, *A*_*i*_, *v*_*i*_, and *D*_*i*_ are the occurrence of δ*x*, time interval, displacement in the time interval, amplitude of the *i*th mode, drift velocity of the *i*th mode, and diffusion coefficient of the *i*th mode, respectively.

## Supplementary information

Supplementary information

clean version of SI

Supplementary video 1

Supplementary video 2
