# Supplementary material for: Rational design using sequence information only produces a peptide that binds to the intrinsically disordered region of p53
Source: Sci Rep. 2019 Jun 28;9:8584. doi: 10.1038/s41598-019-44688-0 (PMC6599006; doi:10.1038/s41598-019-44688-0)
Supplement: Supplementary file 1 — Supplementary information [file 41598_2019_44688_MOESM1_ESM.pdf]

Supplementary Data

Rational design using sequence information only produces a peptide that binds to the intrinsically disordered region of p53

Kiyoto Kamagata[1]*, Eriko Mano[1], Yuji Itoh[1,2], Takuro Wakamoto[3], Ryo Kitahara[4], Saori Kanbayashi[1], Hiroto Takahashi[1], Agato Murata[1,2], and Tomoshi Kameda[5]*

[1]Institute of Multidisciplinary Research for Advanced Materials, Tohoku University, Katahira 2-1-1, Aoba-ku, Sendai 980-8577, Japan

[2]Department of Chemistry, Graduate School of Science, Tohoku University, Sendai 980-8578, Japan

[3]College of Life Sciences, Ritsumeikan University, Kusatsu, 525-8577, Japan

[4]Department of Pharmaceutical Sciences, College of Pharmaceutical Sciences, Ritsumeikan University, Kusatsu, 525-8577, Japan

[5]Artificial Intelligence Research Center, National Institute of Advanced Industrial Science and Technology (AIST), Koto, Tokyo 135-0064, Japan

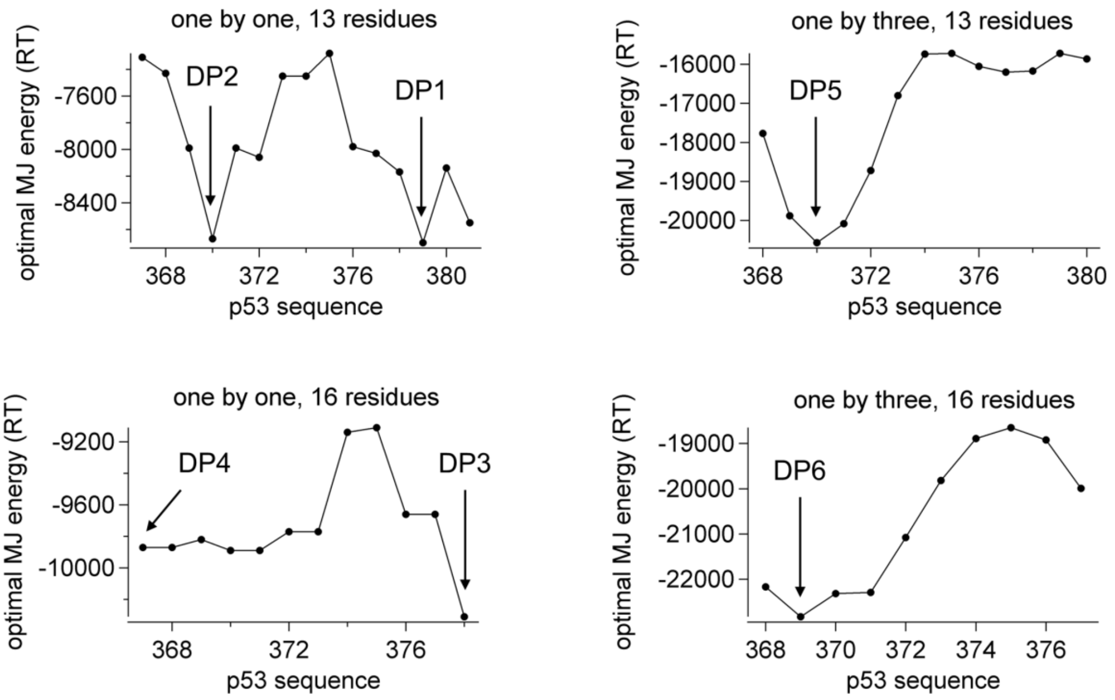

Fig. S1. Designed peptides were selected among the optimal peptide for each part of p53 sequence based on the Miyazawa-Jernigan (MJ) energy. The p53 sequence denotes the initial sequence number used for designing the complementary peptide. Lowest MJ binding energy of 13-residue or 16-residue peptide against p53 sequence was plotted considering one by one or one by three interactions. The arrows represent the designed peptides DP1−6.

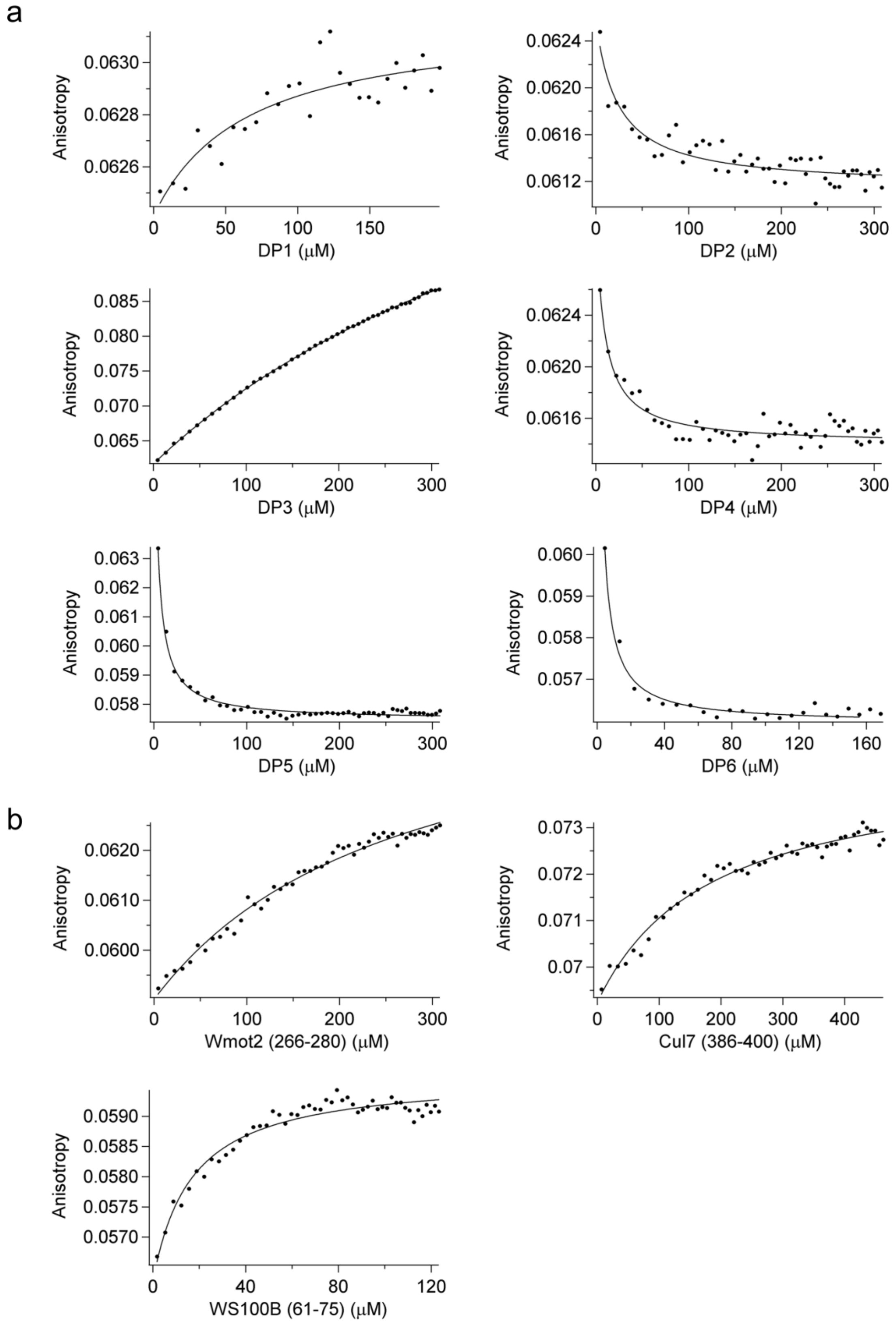

Fig. S2. Titration of designed peptides (a) or peptides from natural proteins [1]. (b) against

the CT domain of p53. Fluorescence anisotropy changes in the CT domain labeled with 6-FAM were monitored upon the addition of the peptides. The black curves were best fitted curves based on the one to one binding model.

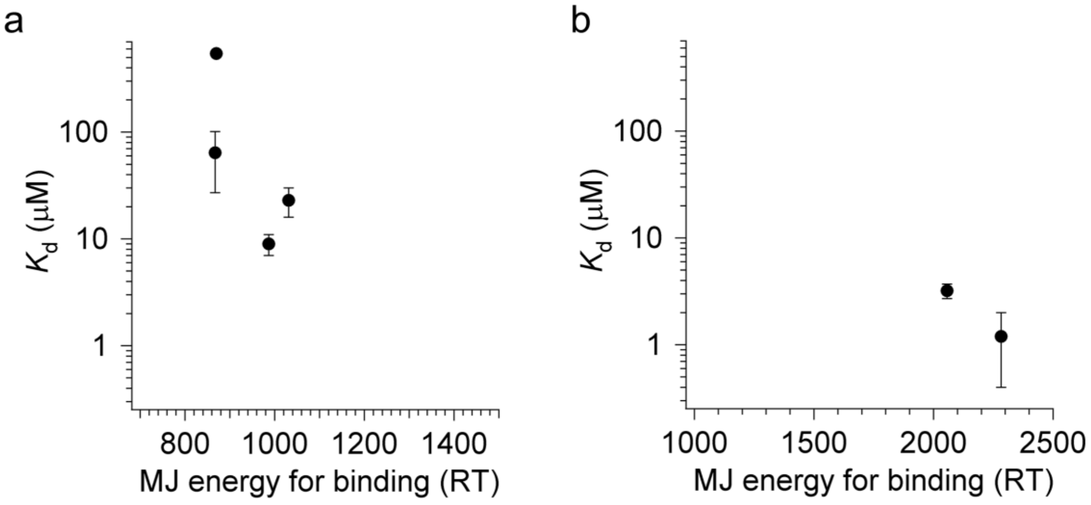

Fig. S3. MJ binding energy correlates with affinity for the designed peptide−CT domain complex. The binding energy was calculated considering one by one residue interaction for DP1−DP4 (a) or one by three residue interaction for DP5 and DP6 (b). The affinity was determined in the absence of KCl by monitoring the fluorescence anisotropy change in the CT domain labeled with 6-FAM. The error is the SEM of the fitting in the titration measurements.

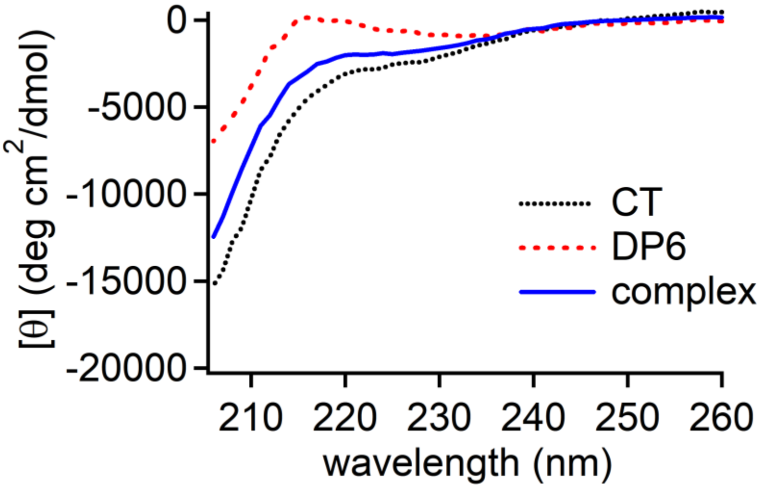

Fig. S4. DP6 and CT peptide of p53 do not form a secondary structure in the complex.

CD spectra of DP6 and CT peptides and the complex.

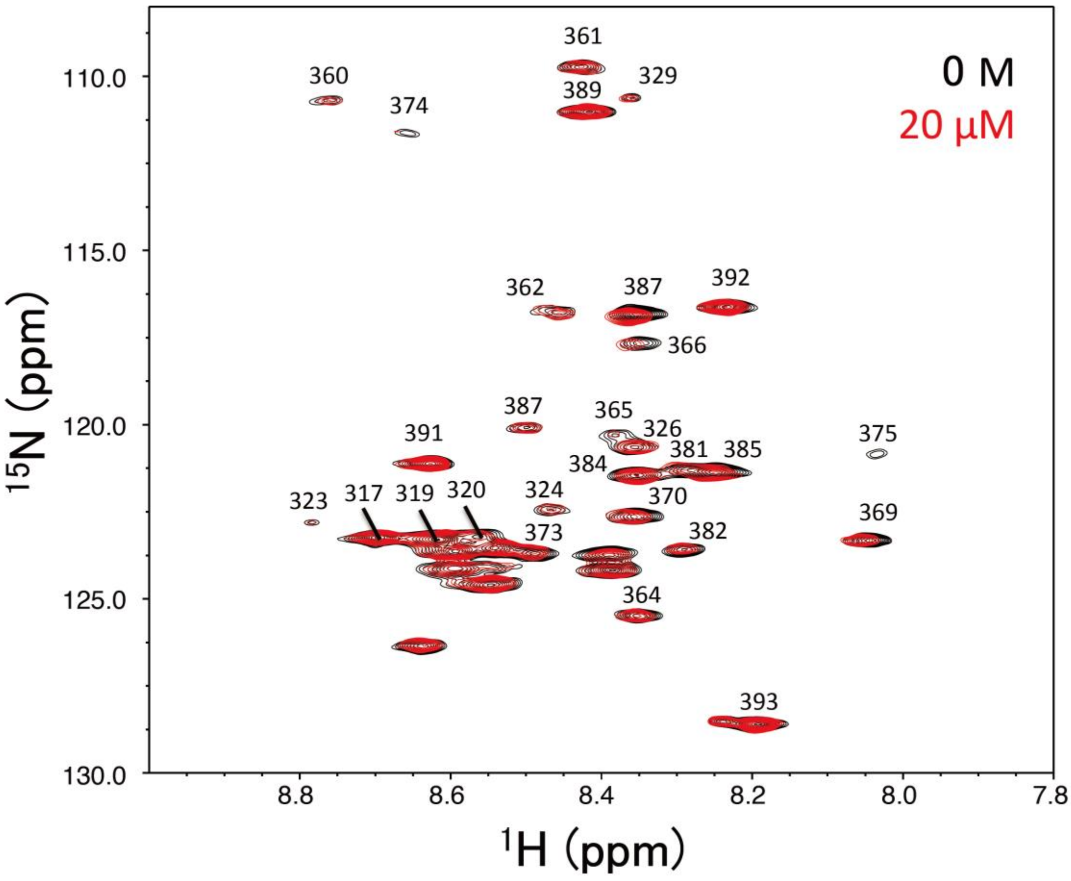

Fig. S5. Binding site of DP6 in p53 was identified using NMR. $^{1}H/^{15}N$-HSQC spectra of ^{15}N-labeled tetrameric p53 (313–393) at 0 μM (black) and 20 μM (red) DP6.

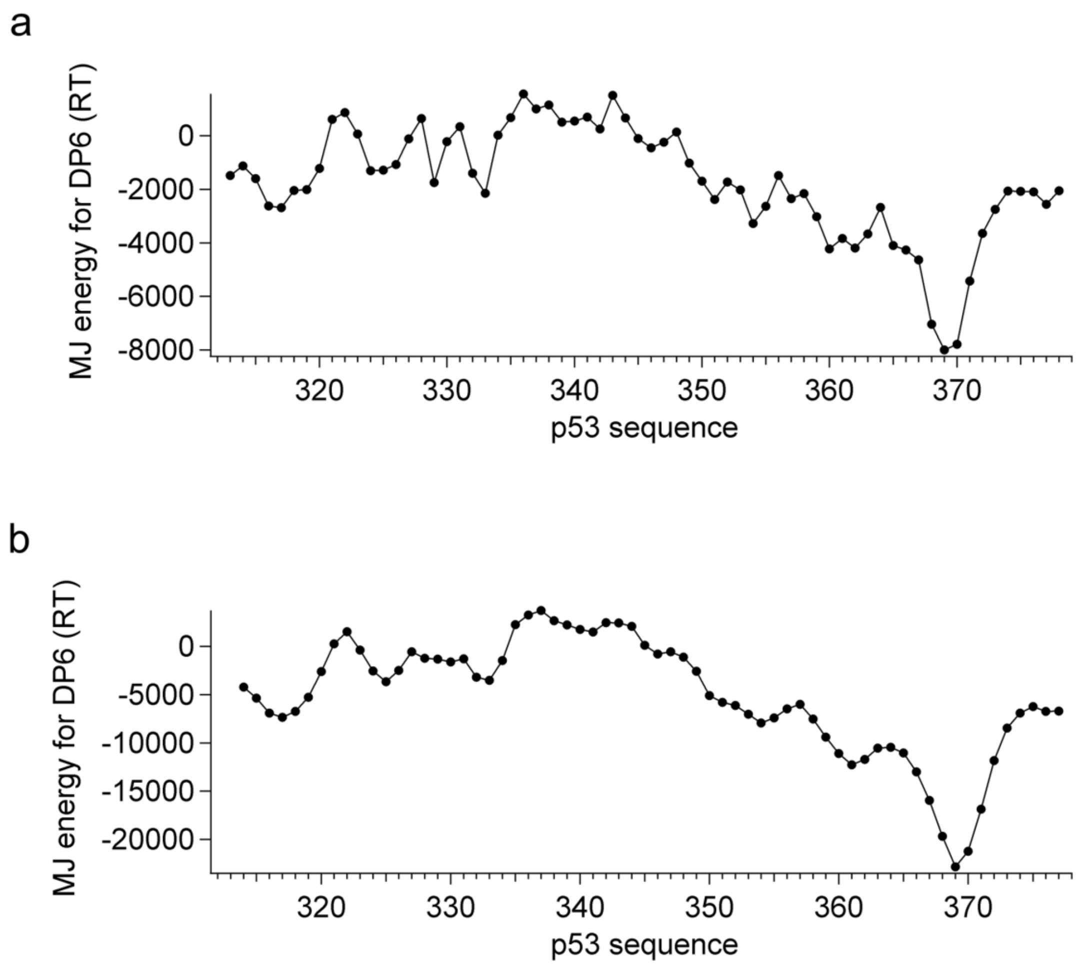

Fig. S6. MJ binding energy of DP6 to p53 is consistent with the binding site identified by NMR and MD simulation. MJ binding energy of DP6 to p53 sequence was calculated considering one by one interaction (a) or one by three interaction (b). MJ energy for DP6 against p53 sequence was calculated. The p53 sequence denotes the initial sequence number used for the calculation. Residue 368–371 in panel (a) and residue 366–372 in panel (b), representing the minimum of MJ energy landscape for binding to DP6, correspond to residue 368–386 and residue 366–387 in Fig. 2 of the main text, respectively.

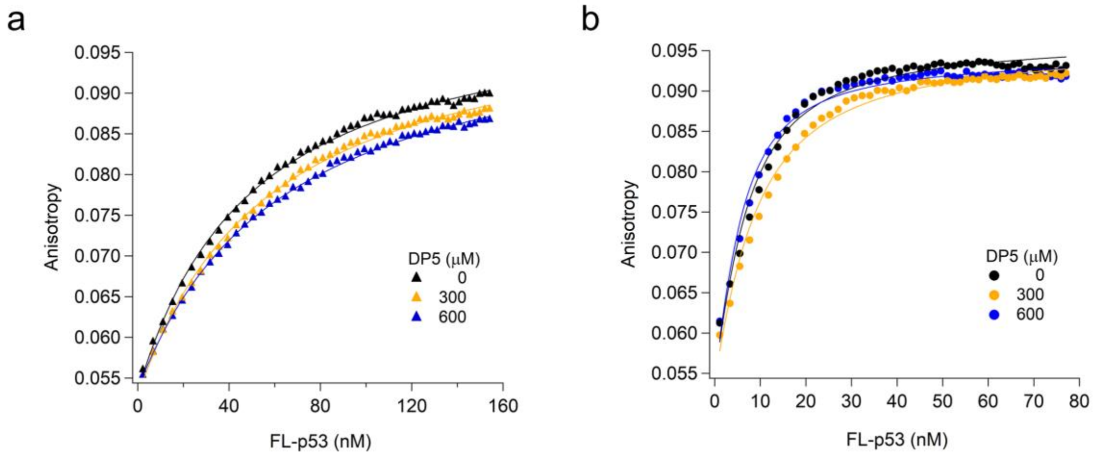

Fig. S7. Designed peptide DP5 does not affect the DNA binding of p53**.** (a) Titration of FL-p53 against nspDNA at various DP5 concentrations. (b) Titration of FL-p53 against spDNA at various DP5 concentrations. Tetramer concentrations are used for p53 mutants. Solid curves are best-fitted curves using Eqs. 1 and 2 in the main text.

Table S1. Dissociation constants of p53 mutants with specific and nonspecific DNAs in the presence of DP6.

DP6 (μM)	DNA	p53 mutant	K_d (μM) [a]
0	p21	FL-p53	7.2 ± 0.4
75	p21	FL-p53	9.2 ± 0.7
150	p21	FL-p53	12.0 ± 0.9
300	p21	FL-p53	20 ± 2
450	p21	FL-p53	22 ± 2
600	p21	FL-p53	21 ± 2
0	random sequence	FL-p53	24 ± 2
75	random sequence	FL-p53	32 ± 3
150	random sequence	FL-p53	57 ± 5
300	random sequence	FL-p53	90 ± 10
450	random sequence	FL-p53	130 ± 20
600	random sequence	FL-p53	160 ± 40
0	random sequence	TetCT	110 ± 20
75	random sequence	TetCT	340 ± 60
150	random sequence	TetCT	n.d.
300	random sequence	TetCT	n.d.
450	random sequence	TetCT	n.d.
600	random sequence	TetCT	n.d.
0	p21	CoreTet	19 ± 3
600	p21	CoreTet	13 ± 3
600	random sequence	CoreTet	n.d.

[a] K_d was determined by fitting the titration curves with Eqs. 1 and 2 in the main text. The error of K_d is the SEM of the fitting. n.d. represents data for which K_d was not determined.

Table S2. Dissociation constants of FL-p53 with specific and nonspecific DNAs in the presence of DP5.

DP5 (μM)	DNA	K_d (μM)[a]
0	p21	6.6 ± 0.4
300	p21	8.5 ± 0.4
600	p21	5.3 ± 0.3
0	random sequence	49 ± 2
300	random sequence	55 ± 2
600	random sequence	61 ± 2

[a] K_d was determined by fitting the titration curves with Eqs. 1 and 2 in the main text. The error of K_d is the SEM of the fitting.

Table S3. Fraction and diffusion coefficient of each sliding mode of FL-p53

[DP6]	Fraction (%)		*D* of each mode ($\mu m^2/s$) ‡		Averaged *D*
(μM)	Fast	Slow	Fast	Slow	($\mu m^2/s$) $
0	61 ± 1	39 ± 2	0.26 ± 0.01	0.032 ± 0.003	0.179 ± 0.005
300	46 ± 2	54 ± 1	0.21 ± 0.01	0.030 ± 0.002	0.092 ± 0.004
600	69 ± 1	31 ± 2	0.19 ± 0.01	0.026 ± 0.004	0.116 ± 0.005

‡ *D* is the diffusion coefficient determined by the displacement analysis. $ *D* is the diffusion coefficient determined by the MSD analysis.

Supplementary movie

Molecular dynamics simulation of DP6 and p53 (313−393) complex.

References

1. Gabizon, R., et al. Specific Recognition of p53 Tetramers by Peptides Derived from p53 Interacting Proteins. *PLoS One* **7**, e38060 (2012).
